# Circadian secretion rhythm of GLP-1 and its influencing factors

**DOI:** 10.3389/fendo.2022.991397

**Published:** 2022-12-02

**Authors:** Chuanfeng Liu, Yuzhao Liu, Yu Xin, Yangang Wang

**Affiliations:** Department of Endocrinology, The Affiliated Hospital of Qingdao University, Qingdao, China

**Keywords:** GLP-1, circadian rhythm, dietary structure, short sleep duration, intestinal flora

## Abstract

Circadian rhythm is an inherent endogenous biological rhythm in living organisms. However, with the improvement of modern living standards, many factors such as prolonged artificial lighting, sedentarism, short sleep duration, intestinal flora and high-calorie food intake have disturbed circadian rhythm regulation on various metabolic processes, including GLP-1 secretion, which plays an essential role in the development of various metabolic diseases. Herein, we focused on GLP-1 and its circadian rhythm to explore the factors affecting GLP-1 circadian rhythm and its potential mechanisms and propose some feasible suggestions to improve GLP-1 secretion.

## Introduction

Glucagon-like peptide-1 (GLP-1) is an incretin mainly secreted by intestinal L cells (1), promoting insulin secretion in a glucose-dependent form. GLP-1 can also produce various non-glycemic effects through the systemic expression of a wide range of GLP-1 receptors (2) such as cardiovascular protection (3),lowering blood pressure (4), regulating lipid metabolism (4), and controlling gastrointestinal motility and delayed gastric emptying. A small amount of GLP-1 expression is found in the nucleus accumbens (5); because peripherally secreted GLP-1 does not cross the blood-brain barrier (6). Hence, only GLP-1 expressed in the nucleus accumbens acts on the central GLP-1R, which might be one of the reasons why GLP-1 can affect cognitive function and mood in addition to suppressing appetite (6). GLP-1 analogs are also approved as first-line drugs for type 2 diabetes and obesity (7).

Circadian rhythms are endogenous biological rhythms with a cycle of approximately 24 hours in organisms, mainly regulated in response to light and darkness changes, and are formed by various transcription factors and promoters that form an autoregulatory feedback loop (8). This feedback system is expressed not only in the supraoptic nucleus of the hypothalamus but also in peripheral tissues such as pancreatic islets, adipose tissue, gastrointestinal tract, liver and skeletal muscle (9, 10). Circadian rhythm stability is closely related to the stability of multiple metabolic pathways (8). However, the artificial lighting used to maintain a constant ambient temperature, sedentary lifestyle, and availability of cheap high-calorie food affects circadian program mechanisms (11). Disruption of circadian rhythms is a risk factor for metabolic disorders and can lead to various metabolic diseases, including impaired insulin secretion (12), abnormal glucose tolerance (12), obesity, and even diabetes (13).

This review focus on GLP-1 and its secretion rhythm as a clue to explore the factors influencing GLP-1 secretion rhythm and the role of exogenous GLP-1-like regulation in GLP-1 rhythm.

## GLP-1 biological rhythm

GLP-1 is an incretin secreted by intestinal L cells. As a link between intestinal endocrine cells and pancreatic β-cells, GLP-1 can regulate insulin secretion in a glucose-dependent manner, and it is jointly responsible for approximately 50% of nutritionally induced insulin secretion with GIP (14). his phenomenon might be related to how L cells are stimulated by food to regulate GLP-1 secretion (15) and the fact that GLP-1 is rapidly hydrolyzed by the DPP-IV enzyme about 2 min after secretion (16). Thus, the temporal rhythm of GLP-1 secretion has not been found for a long time (17). Only in 2009, ola Lindgren et al. used N- and C-terminal directed antisera to measure GLP-1 concentrations after standardized food intake in healthy men and performed the first *in vivo* experiments revealing a temporal difference in GLP-1 secretion and demonstrating that early GLP-1 and GIP release was more pronounced in the morning than in the afternoon (18). A Further, a significant circadian rhythm in GLP-1 secretion was found in an *in vivo* GLP-1 test in response to OGTT in mice (19). Martchenko also identified an important role for the core biological clock gene Arnt1 in regulating time-dependent GLP-1 secretion in intestinal L cells in mice (20). Knockdown of the core biological clock gene Bmal1 in mice and transcriptional analysis of intestinal slices demonstrated that Bmal1 and its downstream target SNARE regulatory proteins are key regulatory proteins in maintaining GLP-1 circadian secretion (21–23). Additionally, Synaptotagmin-7 (24) is now considered a positive regulatory protein of GLP-1.

Furthermore, the intestinal flora regulation of GLP-1 secretion rhythm should not be neglected. The intestinal flora is not only necessary for maintaining the GLP-1 rhythm. For example, the rhythmic secretion of GLP-1 by L cells depends on the homeostasis of the intestinal flora environment (25). It also regulates central GLP-1 sensitivity and systemic metabolic processes through the microbial-gut-brain-liver axis (26, 27). This section will be discussed later.

In summary, GLP-1 has a physiological circadian secretory rhythm mediated by L cells and regulated by various core biological clock genes, as well as the intestinal environment. The homeostasis of this rhythm also plays a crucial role in connecting intestinal endocrine cells and pancreatic β-cells.

## Disruption of GLP-1 secretion rhythm

Besides L-cells’ biological rhythms regulating GLP-1 release, dietary structure, obesity, prolonged light exposure, sleep disorders, and intestinal flora disorders can affect the rhythmic secretion of GLP-1.

### Dietary structure

**Figure 1:**
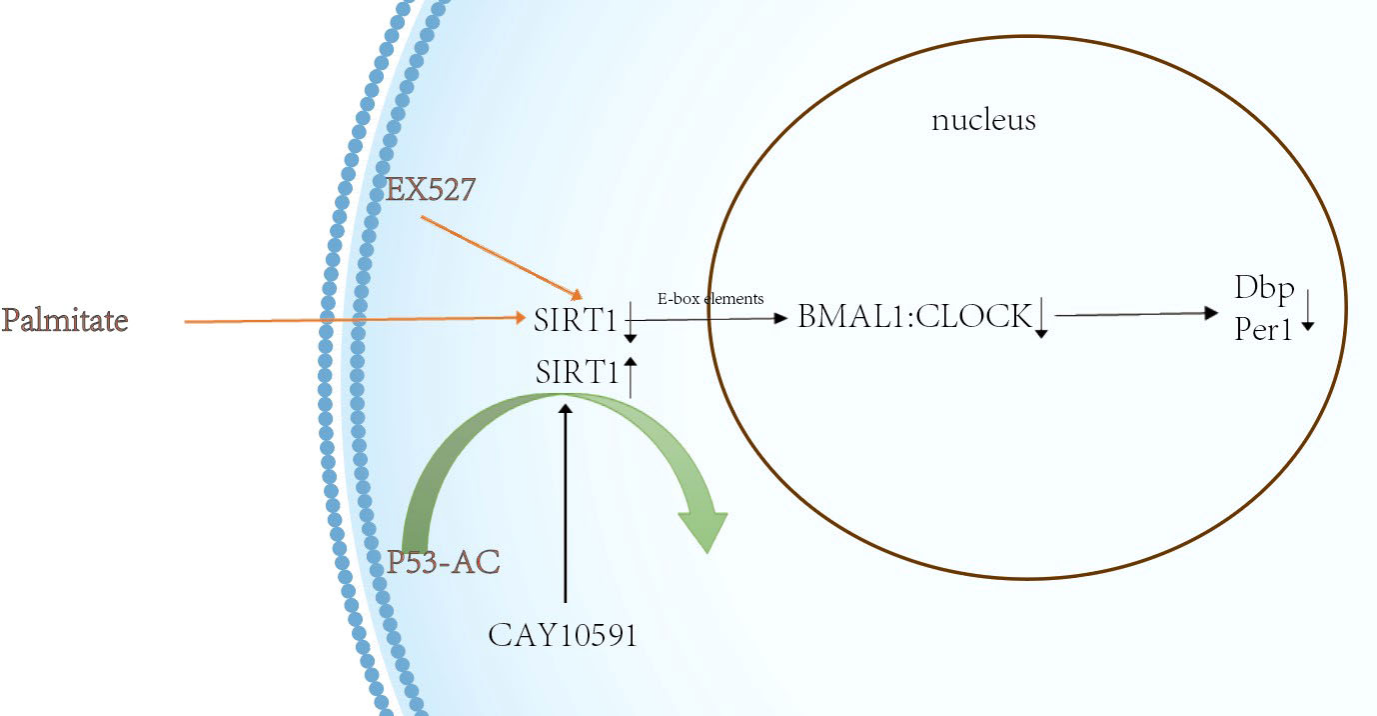
Palmitic acid affects the molecular clock in hepatocytes. SIRT1 can regulate the transcription of CLOCK - and BMAL1 through the promoter E-box element (40), and regulate the expression of Dbp, Per1 and other circadian rhythm genes(black lines). Palmitate can inhibit the expression of rhythm genes by suppressing BMAL1:CLOCK splicing in the form of SIRT1(orange line). EX527 is a SIRT1-specific antibody that produces the same effect as palmitate, have the same inhibitory effect as palmitic acid. CAY10591 can restore SIRT1 activity inhibited by palmitic acid.

High-fat diets alter normal metabolic circadian rhythms in mice (28), and specific high-fat diets do not disrupt biological clock rhythms within the center, but can affect intestinal L-cell and islet β-cell rhythms (29). This might be related to L cells having an independent, autonomous rhythmic clock (30). The *in vitro* culture of the NCI-H716 human intestinal cell line revealed that nutrients such as palmitic acid, oleic acid and meat hydrolysates can stimulate GLP-1 secretion in a dose-dependent manner (31), however, long-term exposure to long-chain saturated fatty acids such as palmitic acid can lead to ceramide accumulation, caspase-3 activation, and increased DNA fragmentation leading to cell death in GLP-1- producing cells (32). It can also induce apoptosis through lipotoxicity in response to the endoplasmic reticulum (33). In contrast, long-chain unsaturated fatty acids such as oleic acid can have cytoprotective effects by reducing ceramide synthesis, attenuating reactive oxygen species (ROS) production, inhibiting caspase-3 activation, and reducing DNA fragmentation (32, 34, 35). Mice fed a high-fat diet, also disrupt L-cell circadian rhythms (36). So, *in vitro* cultures of mouse mGLUTag L cells (37, 38) and mouse assays (38) revealed that palmitate is a key factor affecting L cells as well as eliminating GLP-1 secretion rhythms, even at non-obesogenic doses, interfering with CLOCK : BMAL1 transcriptional activity, increasing Bmal1 transcriptional repression; and resulting in metabolic disorders (39). SIRT1 can regulate the transcription of CLOCK - and BMAL1 through the promoter E-box element (40), and regulate the expression of Dbp, Per1 and other circadian rhythm genes. SIRT1 can be affected by many factors. In hepatocytes, palmitic acid inhibits the splicing of BMAL1 and CLOCK through SIRT1 inhibition, which reduces the expression of hepatocyte genes, including Dbp and Per1 (41)。EX527, the inhibitor of SIRT1, was found to have the same inhibitory effect as palmitic acid. Resveratrol and CAY10591 were found to restore SIRT1 activity inhibited by palmitic acid. (**Figure 1**).

Ketogenesis might be another potential mechanism reducing GLP-1 secretion induced by a high-fat diet, as found by culturing primary intestinal endocrine cells in mice, where ketone bodies can inhibit approximately 40% of basal GLP-1 secretion (42). In clinical trials related to ketogenic diets, some short-term ketogenic diets or exercise resulted in lower fasting and postprandial levels of GLP-1 (43, 44). Nevertheless, another clinical trial in healthy men concluded that ketogenic diets do not affect GLP-1 secretion in humans (45). Moreover, some studies have found that the metabolic changes associated with a long-term ketogenic diet might have gender differences. One study has found that, after weight loss on a ketogenic diet, while basal GLP-1 levels significantly increased in both men and women, postprandial GLP-1 levels appeared significantly higher only in the female group and did not significantly differ in the male group (46). In contrast, other studies did not observe gender differences (47, 48). These differences might be related to the duration of the ketogenic diet, ketone body levels, and the metabolic differences between humans and experimental animals, but more studies are needed to prove this.

A ketogenic diet (KD) is formulated with a high fat proportion and low carbohydrate proportion and is designed to induce ketogenesis. Using unsaturated fatty acids is superior to consuming saturated fatty acids (49). A high-fat diet induces the expression of the ketogenic enzyme mitochondrial 3-hydroxy-3-methylglutaryl-CoA synthase (mhMGCs) in jejunal tissue and the production of functional ketones, which act on the fatty acid/ketone receptor FFAR3 expressed in the small intestinal epithelium to inhibit GLP-1 secretion (42). Additionally, ketone bodies, especially β-hydroxybutyric acid, inhibit inflammatory responses through multiple pathways, including the inhibition of inflammatory vesicles, especially NLRP3 production; ketone metabolism to increase adenosine levels, which are anti-inflammatory through the A1 and A1 receptor pathways; enhanced NADH oxidation; and inhibition of free radical formation (50); by increasing beneficial bacteriophages and reducing Firmicutes, improving the alpha diversity of the flora (51). This might also be why a ketogenic diet inhibits GLP-1 secretion in the short term and can improve GLP-1 in the long term.

Although carbohydrates and fats are the most important pro-secretors of GLP-1, proteins and peptides have recently been found to promote GLP-1. Shimizu’s study in rats showed that whey protein not only increased GLP-1 secretion but also prolonged GLP-1 action by inhibiting DPP-IV enzymatic activity (52). Besides, some plant proteins, such as those found in rice, maize, and peas, can also promote GLP-1 secretion (53). This might be related to various mechanisms such as increased intracellular calcium (54), extracellular signal-regulated kinase 1/2 (ERK1/2), mitogen-activated protein kinase (MAPK), and p38MAPK (55).

### Obesity

Moghadam et al. have found that GLP-1 levels are lower in the obese rat group than in the lean rat group during the first 6 h of the dark cycle and in the middle of the light cycle (56). Meanwhile, postprandial GLP-1 secretion is similarly impaired in obese rats (57). Several clinical studies have found that obese patients have impaired basal and postprandial GLP-1 secretion compared to normal-weight patients (58, 59). Also, lighter-weight patients have higher levels of postprandial GLP-1 secretion compared to normal-weight or obese patients (60). In contrast, a clinical study has found that although obese and overweight patients have higher basal levels of GLP-1 than normal-weight, their secretion loss curves were flatter (61). Unlike the two previous trials, with normal-weight patients who reached standard weight through weight loss, the paradox might be because exercise weight loss alone did not restore normal GLP-1 secretion.

Lipid overload from obesity and validation might explain its effect on GLP-1 secretion. Inflammatory cell infiltration in adipose tissue, muscle, pancreas, and liver due to a saturated fatty acid diet, obesity, and elevated levels of inflammatory cytokines such as tumor necrosis factor, IL-1β, and IL-6 result in persistent chronic low-grade inflammation (62, 63). Notably, for a short time, TNFα promotes GLP-1 secretion to regulate insulin secretion after food intake to maintain glucose homeostasis (64). However, long-term exposure to TNFα impairs GLP-1 secretion (64, 65). Activation of the IL-6 transduction pathway can also increase GLP-1 secretion through the leptin pathway (66, 67). Overall, this mechanisms might be a protective compensatory measure of the organism.

### Long light and short sleep

As shift and night work become more common in modern industrial societies, shorter night shift intervals do not provide sufficient recovery time to adjust circadian rhythms, resulting in poor sleep quality (68), prolonged artificial light exposure, and reduced sleep duration (69). The increase in the incidence of cancer, diabetes, cardiovascular disease, and psychiatric disorders (70–72), might also be related to disruption of sleep-wake rhythms, impaired secretion of melatonin from nighttime light, combined with obesity and a tendency to produce reactive oxygen species (73), which also affects the circadian rhythm of GLP-1 secretion.

Circadian regulation of L-cell activity in rats is highly sensitive to disturbances in circadian rhythms, as continuous light conditions eliminate normal changes in GLP-1 and insulin nutrient-induced responses and significantly impair glucose tolerance (19), Moghadam and his team similarly found that basal levels of GLP-1 were higher in rats under dark conditions (56), and sensitivity was highest (74). In a clinical trial on male volunteers, both sleep and prolonged light exposure interfered with GLP-1 secretion (30). The basal GLP-1 peak occurred at 6 am and was significantly lower after continuous light exposure compared to the normal light exposure group, although the node at which this peak occurred did not change. However, after experiencing continuous light, the postprandial GLP-1 peak increased by 24% compared to the previous one. A clinical trial by Benedict et al. in healthy men showed that patients after acute sleep deprivation had a delayed GLP-1 secretion peak after breakfast compared to normal sleep, despite no significant difference in the area under the total GLP-1 curve, for about 90 min (75).

### Intestinal flora

In recent years, the role of intestinal flora in metabolism has received increasing attention. Dysbiosis is closely associated with various metabolic diseases such as obesity (76), gout (77), NAFLD (78), insulin resistance, diabetes mellitus and its complications (79, 80). Herein, we discuss the effects of the intestinal flora on the rhythmic secretion of GLP-1 and observe the mechanisms of related metabolic diseases from the perspective of GLP-1.

A homeostatic intestinal flora environment is necessary for the rhythmic secretion of GLP-1. Obesity, hyperglycemia and hyperlipidemia reduce the alpha and beta diversity of the intestinal flora (81). In germ-free mice without a 24-hour rhythm of insulin secretion, fecal transplantation returned the insulin rhythm, increased their fasting GLP-1 levels, and demonstrated that *Akkermansia muciniphila* and *Lactobacillus* are positively correlated to GLP-1 regulation (25, 82). This might be related to the glucagon 1-inducible protein P9, which induces GLP-1 secretion by activating GPCR-like downstream signals (83). Additionally, IL-6 deficiency blocks this pathway, demonstrating that *Akkermansia* induces GLP-1 secretion *via* the IL-6-P9 axis and that Lactobacillus can regulate bile acid secretion and increase GLP-1 secretion *via* the bile acid receptor FXR/TGR5 pathway (82). Although the roles of *Firmicutes* and *Bacteroides* in obesity need to be further clarified, they can still regulate GLP-1 secretion, and GLP-1 levels can be increased up to twofold in diet-induced obese patients treated with vancomycin compared to untreated patients (84). *Helicobacter pylori* eradication can also promote GLP-1 secretion and improve glucose metabolism, which may be associated with *Lachnobacterium, Bifidobacterium adolescentis, Coriobacteriaceae*, and other strain alterations (85). Besides, germ-free mice or antibiotic-induced mice can enhance central nervous sensitivity to leptin mediated by GLP-1RA (27).In contrast, mice supplemented with probiotic strains, such as *Lactobacillus*, can promote GLP-1 secretion (86–88). This increased secretion might be caused by reduced TNF-α and IL-6, inhibition of inflammation, antioxidant activity, increased short-chain fatty acid-related GLP-1 secretion, and regulation of bile acid secretion (89, 90).

### Other factors

Current studies have demonstrated significant gender differences in both the structure of the supraoptic nucleus (91), electrophysiological activity (92) and the expression of androgen and estrogen receptors within the nucleus accumbens. Males express higher levels of androgens than females in the supraoptic nucleus, but lower levels of estrogen α receptors (93, 94). The expression levels of these receptors are influenced by circulating hormone levels, representing a direct interaction of gonadotropin levels with the central master clock, leading to sex differences in a wide range of physiological processes controlled by the circadian system, including the HPG axis, the HPA axis, and sleep-wake cycle (95).

Other factors also affect GLP-1 rhythm. For example, growth inhibitory hormone can act on growth inhibitory hormone receptor 5 on L cells to inhibit GLP-1 secretion (96). Knockdown of SCGN, an action-binding regulatory protein, in mice leads to a loss of GLP-1 circadian rhythm in response to glucose, demonstrating that SCGN is an important factor in maintaining GLP-1 circadian rhythm. This may be mediated by SCGN regulating secretory granules (97). The effect of diabetic models on GLP-1 rhythms is currently unclear, but a phase shift in circadian rhythm patterns can be found in high-fat diet/streptozotocin mouse models (98).

Obesity, diet, long light and short sleep, and dysbiosis of the gut flora can promote systemic chronic low-grade inflammation and oxidative stress leading to insulin resistance and increased risk of diabetes (56, 99–102). In recent years, gut flora has also been recognized as an important causative factor for diabetes (103). The GLP-1 and insulin secretion rhythm are consistent in both physiological and pathological states, and multiple factors might explain the pathological mechanisms of insulin resistance and diabetes from another perspective by altering the GLP-1 secretion rhythm by L cells.

## GLP-1 circadian rhythm therapy

Disruption of GLP-1 rhythm leads to disruption of the corresponding insulin secretion rhythm. Therefore, by treating the above-related risk factors, the rhythmic secretion of re-GLP-1 can be restored and glucose metabolism can be improved.

As mentioned above, adequate sleep and a healthy diet such as a ketogenic diet can improve GLP-1 secretion through different mechanisms including inhibition of the inflammatory response and improved flora α diversity. Additionally, exercise is an important tool recommended by the ADA guidelines to prevent and treat obesity in diabetes mellitus patients (104) and can improve patients’ blood glucose levels and insulin resistance (105). Reduction of both insulin resistance after weight loss and chronic low-grade inflammation due to obesity contribute to the rhythmic recovery of GLP-1 levels. Exercise can affect the expression of various circadian rhythm-related genes (106) and influences the expression of the central hypothalamic clock, correlating with the expression of the clock genes per1 and per2 (107). Thomas et al. found that circadian rhythms could be phase-shifted by timed exercise interventions (108). They showed that early morning exercise advanced the melatonin phase, while late evening exercise delayed it. Exercise can also modulate the clock phase in skeletal muscle independent of the central clock (109). Exercise in obese mice under dark conditions increases the abundance of clock core proteins, such as BMAL1 and CLOCK proteins, in skeletal muscle (110). Adipose is an important endocrine tissue in the body, and white and brown adipose tissue are equally circadian (111). Exercise on adipose tissue can similarly regulate glucose and energy metabolism by modulating circadian gene expression in an adipose tissue-mediated manner (109).

However, weight loss through exercise and diet therapy alone does not fully restore rhythmic GLP-1 secretion, and the metabolic changes associated with diet control alone and exercise weight loss are inconsistent. Joaquín et al. showed that, despite a 5% reduction in body weight through diet control, unlike Ghrelin and YY peptide, GLP-1 levels did not change (61). Adam et al. found that after weight loss through a very-low-energy diet (VLED), GLP-1 levels were reduced compared to before weight loss (112). After 8 weeks of a low-energy diet (LED), Sloth similarly found a decrease in GLP-1 levels in subjects (113). In contrast, a decrease in GLP-1 levels was not found with exercise weight loss but rather a trend towards higher postprandial GLP-1 (114, 115). This might be related to epigenetic changes resulting from long-term obesity in patients who have lost weight after obesity. Changes in cellular stress, adipokine secretion, and lipolysis induced by weight loss (87), as well as biological drivers due to imbalances in energy supply and demand (88), contribute to rebound after weight loss. The vagus nerve might also play an important role in reducing GLP-1 secretion (116). The difference between diet and exercise might be because diet weight loss is a reduction in intake and inhibition of nonesterified fatty acids (NEFA), and elevated NEFA levels inhibit GLP-1 secretion (112). This might also be one of the reasons why dietary weight loss is more likely to rebound than exercise weight loss. From this perspective, exogenous supplementation of GLP-1 analogs can restore the autonomous GLP-1 secretion function of L cells (117) and effectively prevent weight loss failure. As the relationship between dysbiosis and metabolic diseases has been gradually studied, treatment by intestinal flora has received increasing attention. As mentioned earlier, antibiotic-induced strain changes can improve GLP-1 secretion rhythm. However, the abuse of antibiotics is not good. Therefore, supplementation with probiotics such as Lactobacillus is recommended to improve the alpha diversity of the intestinal flora (118). Additionally, dietary modification and weight loss treatment can help Firmicutes and Bacteroides abundance decrease, which might also help achieve improved intestinal flora. Nobiletin was found to improve the rhythm of GLP-1 secretion in high-fat-induced mice, and could increase fasting and postprandial GLP-1 levels. This may be related by improving lipid metabolism and modulating the structure of the intestinal flora (119, 120).

Furthermore, GLP-1 analogs, such as liraglutide, dulaglutide, and semaglutide, are now widely used in the clinic to treat patients with diabetes and obesity by various mechanisms, including anti-inflammation, emergency improvement, intestinal flora regulation, appetite suppression *via* the central nervous system, and weight reduction (121–123). Exogenous GLP-1 analog supplementation can restore the GLP-1 physiological secretion rhythm and the circadian rhythm of islet function (117, 124), which might be closely related to the aforementioned metabolic benefits when exogenously supplementing GLP-1 analogs.

We summarized the factors affecting the circadian rhythm GLP-1 and found that exercise can regulate the circadian rhythm (**Tables 1**, **2**). Exercise and its associated weight loss can improve the GLP-1 secretion rhythm and might be more effective in preventing weight regain. However, the effects of diet, and dietary weight loss, are currently controversial. Short-term ketogenic diets are believed to reduce GLP-1 secretion, while long-term ketogenic diets might improve GLP-1 secretion levels, which needs further validation. Meanwhile, long-chain saturated fatty acids, represented by palmitic acid, have an inhibitory effect on circadian rhythms. Additionally, protein, peptides, and supplementation with intestinal probiotics contribute to GLP-1 secretion, while poor lifestyle habits such as long light and short sleep at night can impair GLP-1 secretion levels. Therefore, we recommend a good routine, appropriate exercise, healthy eating habits, and, if necessary, GLP-1 analogs or probiotic supplementation to improve the secretion rhythm.

**Table 1:** Clinical studies affecting GLP-1 secretion.

Factors	Numbers	objects	secrection	reference
TNFα	n=12	human	↓	65
IL-6	n=19	human	↑	67
ketogenic diet	n=13	human	↑	44
ketogenic diet	n=10	human	–	45
loss weight	n=25	human	–	61
VLED	n=32	human	↓	112
LED	n=131	human	↓	113
exercise	n=22	human	↑	114
exercise	n=14	human	↑	115
NEFA	n=32	human	↓	112
liraglutide	n=51	human	↑	117
obese	n=13	human	↓	58
overweight	n=28	human	↓	112
constitutional thinness	n=8	human	↑	60
ketogenic diet	n=15	human	↓	43
VLED	n=95	human	male:basal GLP-1 ↓	46
VLED	n=95	human	femal:postprandial GLP-1 ↑	46
VLED	n=31	human	↑	48
Sleep deprivation	n=8	human	↓	38
sleep deprivation	n=12	human	↓	75

**Table 2:** Basic studies affecting the secretion of GLP-1.

factors	object	Secretion	reference
ketone body	cell	↓	42
Palmitate	GLUTag cell	↓	31
Palmitate	GLUTag cell	↓	32
Palmitate	GLUTag cell	↓	33
Palmitate	GLUTag cell	↓	34
Palmitate	GLUTag cell	↓	37
Palmitate	GLUTag cell	↓	30
oleic acid	GLUTag cell	↑	31
oleic acid	GLUTag cell	↑	32
oleic acid	GLUTag cell	↑	33
oleic acid	GLUTag cell	↑	34
nutrient excess	rat	↓	30
obese	rat	↓	56
obese	rat	↓	57
TNFα	rat	Short term↑	64
TNFα	rat	long term↓	64
IL-6	rat	↑	66
protein	rat	↑	52
dark cycle	rat	↑	56
*Akkermansia*	rat	↑	82
*H. pylori*	rat	↓	85
*Lactobacillus*	rat	↑	86
Somatostatin	rat	↑	96
SCGN	rat	↑	97
exercise		↑	

In this review, we used GLP-1 and its circadian rhythm as a clue to explore the factors influencing the circadian rhythm of GLP-1 and its potential mechanisms and suggested some feasible recommendations to improve the secretory rhythm of GLP-1. This review might also provide some therapeutic recommendations for patients, help clarify the mechanisms of restoring GLP-1 secretion, and further develop relevant in treatments.

## Author contributions

CL: Constructing ideas, reviewing literature, and writing papers. YL, YX: Reviewing literature and providing input. YW: Provide guidance. All authors contributed to the article and approved the submitted version.

## Acknowledgements

We are grateful to all the authors who contributed to this article.

## Conflict of interest

The authors declare that the research was conducted in the absence of any commercial or financial relationships that could be construed as a potential conflict of interest.

## Publisher’s note

All claims expressed in this article are solely those of the authors and do not necessarily represent those of their affiliated organizations, or those of the publisher, the editors and the reviewers. Any product that may be evaluated in this article, or claim that may be made by its manufacturer, is not guaranteed or endorsed by the publisher.

## Abbreviations

GLP-1, Glucagon-like peptide-1; ROS, reactive oxygen species; TNF, tumor necrosis factor α; mhMGCs, mitochondrial 3- hydroxy-3-methylglutaryl-coasynthase; MAPK, mitogen-activated protein kinase; ERK1/2, extracellular signal-regulated kinase ½; KD, ketogenic diet; SCGN, Recombinant Secretagogin; LED, low-energy diet; VLED, very-low-energy diet; NEFA, nonest-erified fatty acids; OGTT, oral glucose tolerance test.
